# Psychometric properties of the Chinese version of the school refusal assessment scale–revised in a clinical sample of adolescents and their caregivers with depressive disorders

**DOI:** 10.3389/fpsyt.2026.1859186

**Published:** 2026-07-28

**Authors:** Yulu Wang, Zhaoqian Yao, Qingqing Zhang, Yixin Xiong, Xuebing Huang, Ying Qian

**Affiliations:** 1 Peking University Sixth Hospital, Peking University Institute of Mental Health, NHC Key Laboratory of Mental Health (Peking University), National Clinical Research Center for Mental Disorders (Peking University Sixth Hospital), Beijing, China; 2 The Chinese University of Hong Kong, Hong Kong, Hong Kong SAR, China; 3 The Second Affiliated Hospital of HeNan Medical University, Henan, China; 4 Tsinghua University, Beijing, China

**Keywords:** Chinese adolescents, clinical sample, confirmatory factor analysis, cross-cultural assessment, cross-informant discrepancy, depressive disorders, functional analysis, intraclass correlation coefficient

## Abstract

School refusal behavior (SRB) is a prevalent and functionally heterogeneous problem among children and adolescents that can lead to serious academic, social, and psychological consequences. The School Refusal Assessment Scale–Revised (SRAS-R) is the most widely used instrument for identifying the functional motivations underlying school refusal, yet its psychometric properties have not been examined in Chinese clinical populations. The present study aimed to translate and culturally adapt the SRAS-R into Chinese and to evaluate its psychometric properties in a clinical sample of adolescents with depressive disorders. A total of 171 adolescent outpatients (age range 12–19 years; *M* = 15.5, *SD* = 1.90; 67.3% female) diagnosed with DSM-5 depressive disorders and meeting criteria for school refusal behavior completed both child and parent versions of the Chinese SRAS-R. Confirmatory factor analysis (CFA) using diagonally weighted least squares estimation was conducted. After removing Items 20 and 24, the four-factor model yielded strong CFI and RMSEA values for both parent (CFI = 0.99, RMSEA = 0.021, SRMR = 0.094) and child reports (CFI = 0.98, RMSEA = 0.028, SRMR = 0.097), although SRMR values were marginally above the prespecified threshold. Internal consistency reliability ranged from marginal to good across subscales (Cronbach’s *α*: parent = 0.664–0.815; child = 0.700–0.864), with parent-reported Factor 4 showing the weakest reliability. Factor 1 (avoidance of aversive school situations) obtained the highest mean scores for both informants, consistent with the depression-related negative affectivity characteristic of this clinical sample. Cross-informant discrepancy analyses revealed that children reported significantly higher scores than parents on Factor 2 (escape from social/evaluative situations; *p* = .017, *d* = 0.18) and Factor 4 (pursuit of tangible reinforcement; *p <.*001, *d* = 0.27), suggesting that parents may underestimate internally driven motivations. Intraclass correlation coefficients indicated fair to good parent–child agreement (ICC = 0.45–0.62), with the highest agreement for Factor 1 and the lowest for Factor 4. The findings provide initial internal-structure and reliability evidence for the Chinese SRAS-R in this single-site clinical sample and underscore the need for multi-informant assessment, while future studies should examine convergent, discriminant, criterion-related, and predictive validity in more diverse samples.

## 1 Introduction

School refusal behavior (SRB) refers to a child-motivated refusal to attend school or difficulty remaining in class for an entire school day (1, 2). SRB is distinct from truancy, school withdrawal, and school exclusion in that it is driven by the child’s emotional distress rather than conduct problems, family decisions, or institutional actions (3–5). With a prevalence of approximately 28–35% among school-aged youth (5, 6), SRB peaks during key developmental transitions and has been further exacerbated by the COVID-19 pandemic (1, 7). Persistent SRB produces severe consequences across multiple domains, including internalizing symptoms (anxiety, depression, somatic complaints), externalizing problems (academic deterioration, family conflict, delinquency), and long-term occupational and social difficulties in adulthood (1, 5, 8–11).

**Figure 1:**
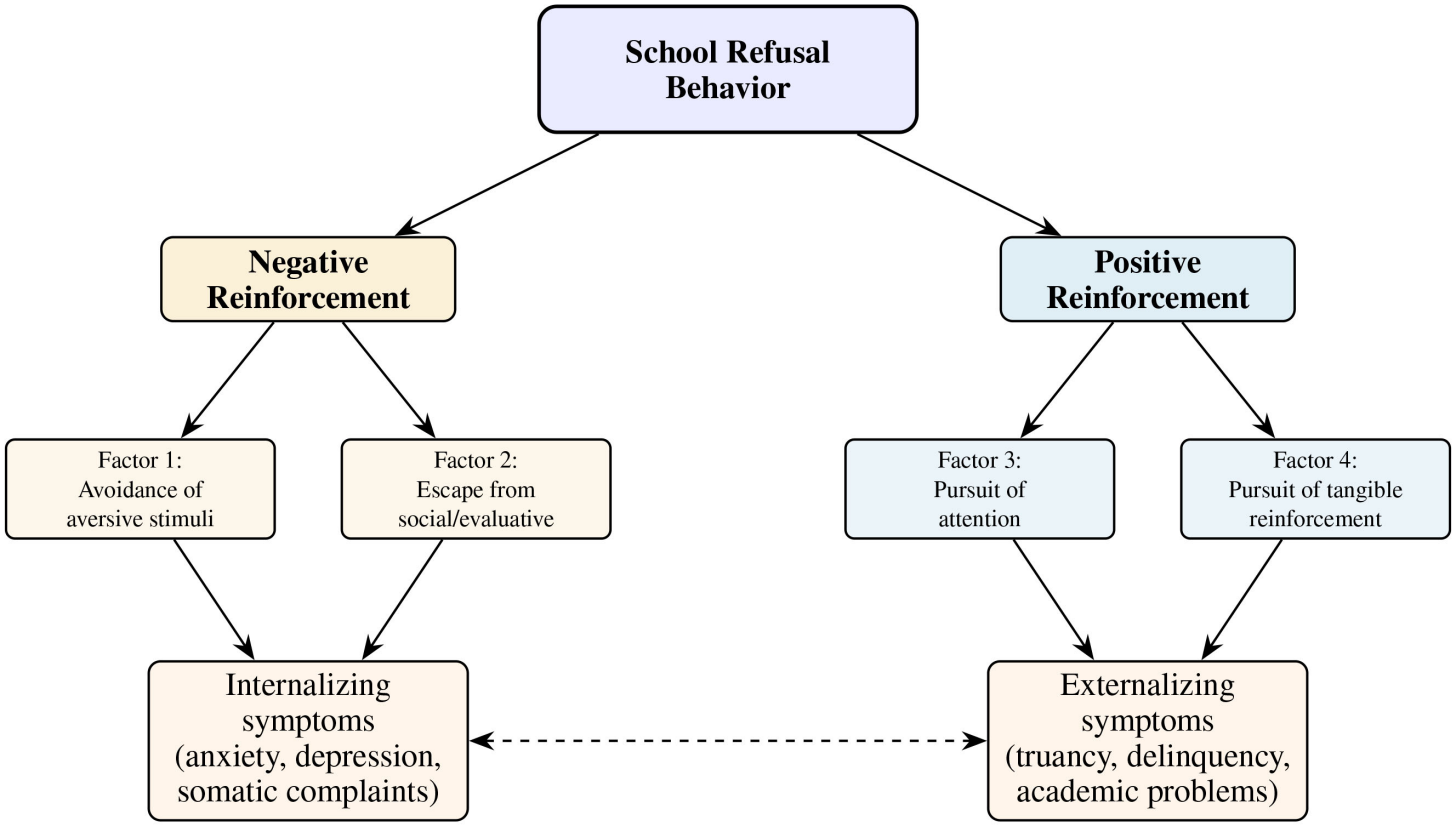
Theoretical framework of the functional model of school refusal behavior (2, 16). School refusal behavior is hypothesized to be maintained by four functional conditions organized along two reinforcement dimensions: negative reinforcement (avoidance of aversive stimuli and escape from social/evaluative situations) and positive reinforcement (pursuit of attention from significant others and pursuit of tangible reinforcement outside school). Each functional profile is associated with distinct patterns of internalizing and externalizing symptomatology. Color-blindfriendly blue/orange shading and direct text labels are used so the figure does not rely on color alone.

A key characteristic of SRB is its functional heterogeneity: individuals with similar observable behaviors may differ substantially in the functions maintaining those behaviors (12, 13). Traditional nosological categories (e.g., “school phobia,” “truancy”) have been criticized for limited clinical utility (14, 15). In contrast, functional approaches focus on the maintaining variables that drive the behavior, regardless of its topographical presentation (13, 16). To address the need for standardized functional assessment, Kearney and Silverman (16) developed the School Refusal Assessment Scale (SRAS), later revised as the 24-item SRAS-R (2). The SRAS-R functional model (see **Figure 1**) posits that SRB is maintained by four reinforcement contingencies: (1) avoidance of school-related stimuli provoking negative affectivity, (2) escape from aversive social and/or evaluative situations, (3) pursuit of attention from significant others, and (4) pursuit of tangible reinforcement outside school. The first two factors reflect negative reinforcement, whereas the latter two reflect positive reinforcement (13). The SRAS-R includes parallel parent and child versions, and combined scores guide identification of the primary functional profile driving SRB (1).

The relationship between SRB and depressive disorders is well established but clinically complex. Depression is among the most common comorbidities in youth with SRB (8, 17, 18), and SRB frequently serves as a presenting complaint in adolescents seeking treatment for depression (9, 19). Depressive symptoms may contribute to school refusal through low motivation, anhedonia, sleep disturbance, fatigue, hopelessness, somatic complaints, and increased sensitivity to aversive school experiences (8, 18). At the same time, prolonged absence from school may worsen depression by reducing peer contact, academic mastery, daily structure, and opportunities for positive reinforcement (5, 20). This bidirectional pattern makes function-based assessment especially important: two adolescents with depressive disorders may both refuse school, but one may be primarily avoiding overwhelming negative affect at school, whereas another may be escaping social evaluation or seeking caregiver proximity.

Cross-cultural assessment is also central to evaluating SRB. Measures developed in one linguistic and educational context may not operate equivalently in another because school attendance is shaped by local expectations about achievement, family obligation, teacher authority, peer relationships, and acceptable expressions of distress (21–23). In China, strong academic expectations, high-stakes examinations, and family involvement in educational decision-making may alter how adolescents and caregivers interpret school-related avoidance, social-evaluative fear, and tangible reinforcement outside school (24–26). The prevalence of depressive symptoms among Chinese adolescents has been estimated at 19.9% (27), yet no study has examined the psychometric properties of the SRAS-R in a Chinese clinical sample of adolescents with depressive disorders. Existing Chinese evidence is limited to a general adolescent population (6), leaving unclear whether the instrument retains its structure in clinical settings where SRB is often more severe, impairing, and functionally complex.

### 1.1 Cross-cultural validation of the SRAS-R

The SRAS-R has been translated and validated in numerous countries, providing an increasingly broad evidence base for the cross-cultural applicability of the four-factor functional model. In the original English validation work, the revised scale showed support for the four functional dimensions in both parent and child versions, although subsequent CFA work also identified weaker performance for some Factor 4 items, especially Items 20 and 24 (2, 28). In a diverse American sample that included youth referred through legal systems, Haight et al. (29) replicated the four-factor structure and extended evidence for the scale to more heterogeneous attendance-problem presentations, including truancy-related cases. Spanish-language research has provided some of the strongest cross-cultural evidence: Gonzálvez et al. (30) examined the Spanish version in a large community sample of children and reported good factorial validity and measurement invariance across gender and age groups, while Gonzálvez et al. (31) validated a Chilean version among 2,678 adolescents and found that the four-factor model showed the best fit and remained invariant across gender and age. The German SRAS-R also demonstrated adequate internal consistency and convergent validity, although weaker Factor 4 indicators again suggested that tangiblereinforcement items may be more sensitive to contextual interpretation (32). Additional adaptations in Norwegian, Turkish, French, Dutch, and Japanese contexts have further supported the clinical and educational relevance of function-based assessment while also showing that item functioning and informant agreement can vary across cultural and service contexts (33–37).

Taken together, these international studies suggest several important patterns. First, the four-factor model generally provides a better representation of school refusal functions than broader two-factor negative- versus positive-reinforcement models (28–31). Second, reliability has typically been acceptable to good for the negative-reinforcement and attention-seeking dimensions, whereas the tangible-reinforcement dimension has shown more variable reliability and weaker item loadings in some samples (28, 32, 38). Third, evidence for measurement invariance in Spanish and Chilean samples indicates that the SRAS-R can support meaningful group comparisons when carefully adapted and tested (30, 31). Finally, the recent validation of the SRAS-R in a general adolescent population in China provides important initial evidence for Chinese-language use (6), but it does not address whether the same functional structure applies in psychiatric clinical settings where school refusal is embedded in depressive symptomatology, greater impairment, and potentially different parent–child reporting patterns. The present study therefore adds value by testing the Chinese SRAS-R in a clinical sample of adolescents with depressive disorders, evaluating both parent and child reports, and examining whether previously noted Factor 4 difficulties also emerge in this cultural and clinical context.

### 1.2 SRAS-R in clinical populations

The SRAS-R has been applied to specific clinical populations, including children with autism spectrum disorder (39, 40), youth with ADHD (38, 41–43), and adolescents with low academic performance and specific learning disorders (44). Research has also examined associations between SRAS-R profiles and emotional regulation, personality traits, self-esteem, and affective profiles (45–48). These studies provide evidence for the construct validity of the functional model across diverse clinical presentations. Importantly, function-based interventions guided by SRAS-R profiles have been shown to effectively reduce SRB and associated psychopathology (49–51), with metaanalytic evidence supporting the efficacy of function-based treatments (52, 53).

### 1.3 Cross-informant discrepancy in child and adolescent assessment

The multi-informant approach to assessing child and adolescent psychopathology is well established (54, 55). Research consistently demonstrates that different informants (e.g., parents, children, teachers) provide only modestly correlated reports, with meta-analytic correlations typically in the range of *r* = .20–.30 for parent–child agreement on internalizing problems (54). These discrepancies are not merely measurement error; they reflect genuine differences in observational access, contextual knowledge, and reporting biases (55, 56).

In the context of SRB assessment, cross-informant discrepancies are particularly relevant because internal motivations for school refusal may be differentially accessible to parents and children. Children may be more aware of their own social anxieties and subjective distress, whereas parents may have better insight into observable behavioral patterns and external contingencies (2). Despite the theoretical importance of cross-informant agreement for the SRAS-R, few studies have systematically examined parent–child discrepancies using methods beyond simple correlations. The present study addresses this gap by employing paired-samples *t*-tests to detect systematic mean-level differences and intraclass correlation coefficients (ICC) to assess absolute agreement between informants across the four SRAS-R factors.

### 1.4 The present study

Taken together, prior research supports the functional model of SRB and the broad cross-cultural utility of the SRAS-R, but three gaps remain. First, the Chinese SRAS-R has not been evaluated in a psychiatric clinical sample, particularly among adolescents with depressive disorders. Second, the performance of potentially problematic Factor 4 items has not been examined in this Chinese clinical context. Third, limited evidence is available regarding parent–child agreement on SRAS-R functional profiles in depressed adolescents, despite the clinical importance of multi-informant assessment.

The present study aimed to address these gaps by translating and culturally adapting the SRAS-R into Chinese and evaluating its psychometric properties—including factorial validity, internal consistency reliability, descriptive statistics, inter-factor correlations, and cross-informant discrepancy—in a clinical sample of adolescents diagnosed with depressive disorders who exhibit school refusal behavior. Beyond evidence based on internal structure, the study also considered validity evidence from response processes during cultural adaptation and from theoretically expected relations among SRAS-R factors and informants. Based on prior validation studies (2, 6, 28, 30–32), we hypothesized that:

(H1) The four-factor structure of the SRAS-R would be confirmed in the Chinese clinical sample, with the four-factor model showing superior fit compared to a two-factor (negative vs. positive reinforcement) model.

(H2) Consistent with prior findings by Kearney (28), a four-factor model excluding Items 20 and 24 would show improved model fit relative to the original 24-item specification.

(H3) Internal consistency reliability would be acceptable to good for all subscales (*α* ≥.70).

(H4) Inter-factor correlation patterns would be consistent with theoretical expectations, with stronger associations among negative reinforcement factors (Factors 1 and 2) than between negative and positive reinforcement factors.

(H5) Factor 1 (avoidance of aversive stimuli provoking negative affect) would be the most endorsed function in this clinical sample of depressed adolescents, given the centrality of negative affectivity in depressive disorders.

(H6) Cross-informant discrepancy analyses would reveal systematic differences between parent and child ratings, with parents potentially underestimating internally driven motivations (e.g., social/evaluative anxiety) relative to adolescents’ self-reports.

## 2 Methods

### 2.1 Participants and procedure

**Figure 2:**
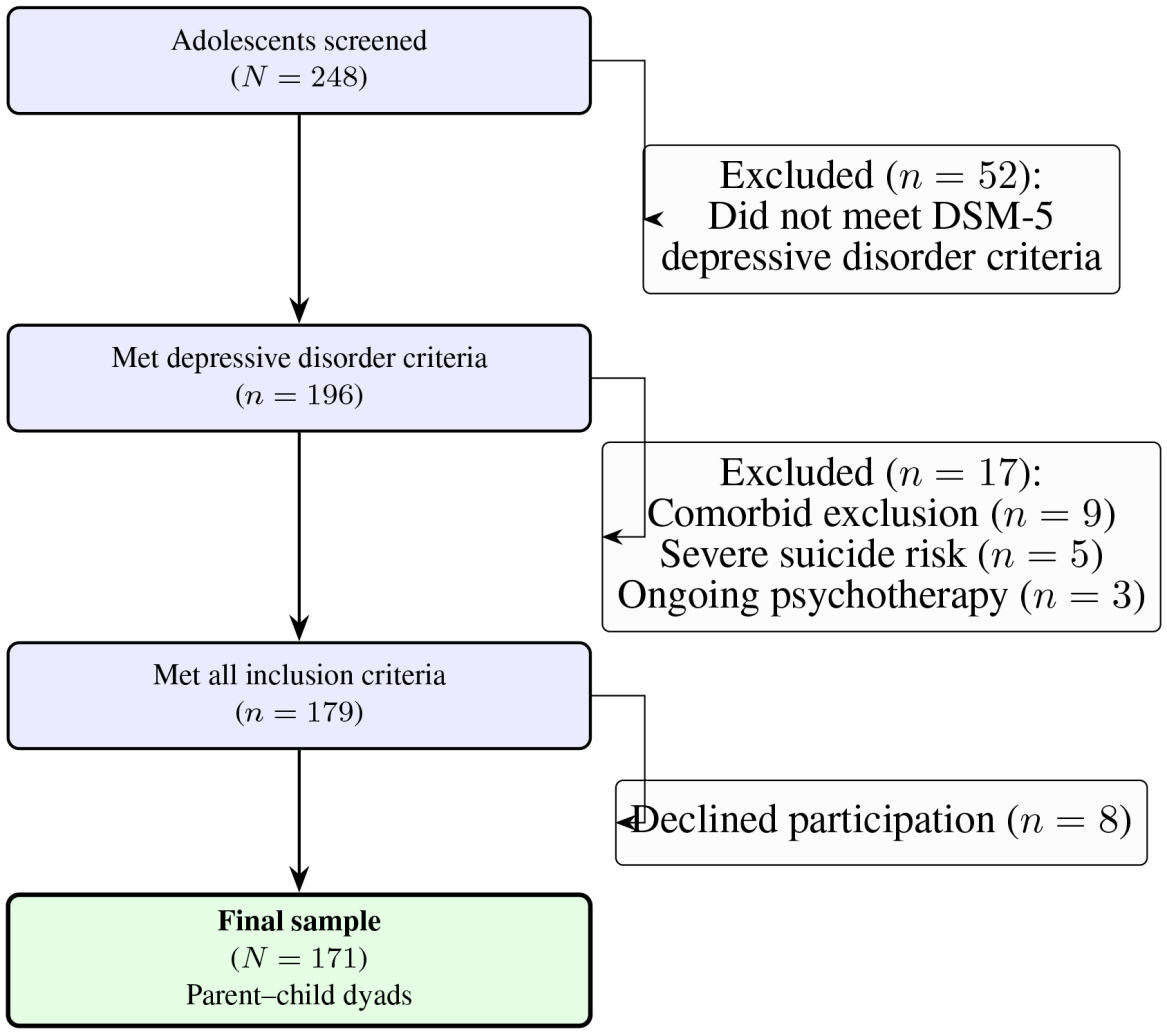
Participant flow diagram showing the screening, eligibility assessment, and enrollment process. A total of 248 adolescents were initially screened, with 171 parent–child dyads meeting all inclusion criteria and completing study measures.

Participants were 171 consecutive adolescent outpatients recruited from the Department of Psychiatry and Psychology at a general hospital in Beijing, China. The recruitment and screening procedure is illustrated in **Figure 2**. Eligible participants met the following inclusion criteria: (a) DSM-5 diagnostic criteria for a depressive disorder (major depressive disorder or persistent depressive disorder), as determined by licensed clinicians using structured clinical interviews; (b) Berg’s (3) criteria for school refusal, including persistent difficulty attending school, marked emotional distress (e.g., excessive fearfulness, temper outbursts, or somatic complaints) when facing school attendance, remaining at home during school hours with parental knowledge, and absence of significant antisocial behavior; (c) age between 12 and 19 years; and (d) school attendance below 85% in the two weeks prior to the clinical visit.

Exclusion criteria included: (a) current or past DSM-5 diagnosis of organic mental disorders, schizophrenia spectrum disorders, schizoaffective disorder, bipolar disorder, attention-deficit/hyperactivity disorder, or other severe psychiatric conditions (57, 58); (b) severe suicide risk as assessed by the treating clinician; (c) severe or unstable physical illness; and (d) current engagement in systematic psychotherapy for depression.

The study protocol was reviewed and approved by the Medical Ethics Committee of Peking University Sixth Hospital (Approval Number: 2025-103-02). All procedures were conducted in accordance with the Declaration of Helsinki and applicable national regulations for research involving minors and clinical populations.

Before participant recruitment, the SRAS-R was translated and culturally adapted into Mandarin Chinese following established cross-cultural adaptation guidelines (21–23, 59). The process included independent forward translation by two bilingual researchers with expertise in clinical psychology and psychometric assessment, reconciliation of the two forward translations by the research team, independent back-translation by a bilingual translator who was blinded to the original English scale, and expert review by the research team and a panel of child and adolescent psychiatry experts. Equivalence between the original and Chinese versions was evaluated by comparing the back-translation with the original SRAS-R item by item, with attention to semantic equivalence, conceptual equivalence, informant perspective, and cultural appropriateness of school-related examples. Discrepancies were resolved through consensus discussion, and the preliminary Chinese version was pilot tested with adolescents and parents to confirm item comprehension before use in the validation sample.

Potential participants were identified during routine outpatient visits by licensed child and adolescent psychiatrists or clinical psychologists. After the clinical assessment, a trained member of the research team briefly introduced the study to eligible families, emphasizing that participation was voluntary, would not affect clinical care, and could be discontinued at any time without penalty. Families who expressed interest received written and verbal information about the study purpose, procedures, potential risks and benefits, confidentiality protections, and data use. Legal guardians provided written informed consent, and adolescents provided written assent before any research questionnaire was administered.

Questionnaires were completed on site after the consent and assent process. Adolescents and caregivers completed the child and parent versions of the Chinese SRAS-R separately in quiet private rooms or separated areas of the clinic to reduce mutual influence. Research staff remained available to clarify procedural questions but did not guide responses or interpret item content. Caregivers also completed the sociodemographic questionnaire. Each questionnaire was identified by a study code rather than personal identifiers; identifying information was stored separately from response data and accessible only to authorized research personnel. Completed paper questionnaires were kept in locked cabinets, and electronic data were stored in password-protected files for analysis.

### 2.2 Sample characteristics

The final sample comprised 171 adolescents with a mean age of 15.5 years (*SD* = 1.90; range = 12–19). The majority were female (67.3%, *n* = 115), with 32.7% male (*n* = 56). Regarding grade level, 42.1% (*n* = 72) were in junior high school (grades 7–9), 49.7% (*n* = 85) were in senior high school (grades 10–12), and 8.2% (*n* = 14) were at other educational levels. At the time of assessment, 16.4% (*n* = 28) were attending school regularly, 17.0% (*n* = 29) had intermittent attendance, 33.3% (*n* = 57) were on leave, and 33.3% (*n* = 57) were suspended from school—indicating that two-thirds of the sample was entirely absent from school, reflecting the clinical severity of SRB in this population. The majority of responding caregivers were mothers (72.5%, *n* = 124). Parental educational attainment was relatively high, with 66.1% of fathers and 69.6% of mothers holding a university degree or higher. The majority of families were married (86.5%, *n* = 148), and 57.9% (*n* = 99) of participants were only children. Full sociodemographic characteristics are presented in **Table 1**.

**Table 1:** Sociodemographic characteristics of the sample (*N* = 171).

Variable	n (%)/M (SD)
Child characteristics	
Age (years) Gender	15.5 (1.90)
Male	56 (32.7%)
Female Grade level	115 (67.3%)
Junior high school	72 (42.1%)
Senior high school	85 (49.7%)
Other Current school status	14 (8.2%)
Regular attendance	28 (16.4%)
Intermittent attendance	29 (17.0%)
On leave	57 (33.3%)
Suspended	57 (33.3%)
Only child	99 (57.9%)
Caregiver and family characteristics Responding caregiver	
Mother	124 (72.5%)
Father	47 (27.5%)
Father’s age at child’s birth (years)	31.6 (4.83)
Mother’s age at child’s birth (years)	29.9 (4.03)
Father education ≥ university	113 (66.1%)
Mother education ≥ university Marital status	119 (69.6%)
Married	148 (86.5%)
Divorced	12 (7.0%)
Other (widowed/separated) Monthly income per capita (RMB)	11 (6.5%)
>10,000	106 (62.0%)
5,001–10,000	42 (24.6%)
1,001–5,000	23 (13.5%)

### 2.3 Measures

#### 2.3.1 School refusal assessment scale–revised

The SRAS-R (2) is a 24-item self-report instrument designed to assess four functional conditions maintaining SRB. Items are rated on a 7-point Likert scale ranging from 0 (*never*) to 6 (*always*). Six items load onto each of the four subscales: Factor 1 (avoidance of school-related stimuli that provoke negative affectivity; Items 1, 5, 9, 13, 17, 21), Factor 2 (escape from aversive social and/or evaluative situations; Items 2, 6, 10, 14, 18, 22), Factor 3 (pursuit of attention from significant others; Items 3, 7, 11, 15, 19, 23), and Factor 4 (pursuit of tangible reinforcement outside of school; Items 4, 8, 12, 16, 20, 24). Subscale scores are computed as the mean of the constituent items, with higher scores indicating a stronger functional motivation for school refusal in that domain. Both child and parent versions are available, with parallel item content adapted for the respective informant perspective. The SRAS-R has demonstrated adequate psychometric properties across multiple cultural contexts (6, 28, 30–32).

#### 2.3.2 Translation and cultural adaptation

The Chinese adaptation of the SRAS-R followed established guidelines for cross-cultural instrument translation (21–23, 59). The translation process consisted of five stages. First, the original English parent and child versions were independently translated into Mandarin Chinese by two bilingual researchers with expertise in clinical psychology and psychometric assessment. Second, the research team compared the two forward translations and reconciled wording differences through structured discussion to produce a unified preliminary Chinese version. Third, an independent bilingual translator who had not seen the original instrument back-translated the reconciled Chinese version into English. Fourth, the research team and a panel of child and adolescent psychiatry experts compared the back-translation with the original English version item by item. Items were reviewed for semantic equivalence, conceptual equivalence, cultural appropriateness, and consistency between parent and child forms; any discrepancies were resolved by consensus while preserving the intended functional meaning of each item. Fifth, the preliminary Chinese version was administered to a small convenience group of adolescents (*n* = 15) and their parents to assess item comprehension, response process validity, and cultural appropriateness (60). The pilot phase was limited to cognitive and linguistic review; it was not used to estimate reliability, factor structure, or other psychometric parameters. Minor wording adjustments were made based on pilot feedback to ensure clarity while preserving the original item content and intent.

#### 2.3.3 Sociodemographic questionnaire

A researcher-designed sociodemographic questionnaire collected information on child age, gender, grade level, current school attendance status, only-child status, parental age at the child’s birth, parental education level, family marital status, number of siblings, and monthly family income per capita.

### 2.4 Data analysis

All analyses were conducted separately for parent and child reports to compare psychometric properties across informants. Descriptive statistics, including means and standard deviations, were calculated for all SRAS-R subscales. Analyses were conducted using JASP (Version 0.13.1; 61). To make the item-removal decision transparent, standardized loadings for Items 20 and 24 in the 24-item ordinal DWLS model were re-estimated from the matched item-level dataset using Python 3.9 and semopy 2.3.11 with all 24 SRAS-R items specified as ordinal indicators.

Confirmatory factor analysis (CFA) was performed using diagonally weighted least squares (DWLS) estimation, which is recommended for ordinal data and has been shown to produce more accurate parameter estimates and standard errors than maximum likelihood estimation when applied to Likert-type scales (62–65). The following fit indices and their respective cutoff criteria were used to evaluate model fit: comparative fit index (CFI ≥ 0.96), root mean square error of approximation (RMSEA ≤ 0.08), standardized root mean square residual (SRMR ≤ 0.09), and the chi-square test of model fit (66–68). Because SRMR cutoffs are less firmly established for ordinal CFA with DWLS estimation, SRMR values were interpreted alongside CFI, RMSEA, chi-square tests, and the substantive interpretability of parameter estimates rather than as a single decisive criterion.

Three CFA models were tested sequentially: (a) a two-factor model collapsing the four subscales into negative reinforcement (Factors 1 and 2) and positive reinforcement (Factors 3 and 4) dimensions, (b) the original four-factor model with all 24 items, and (c) a four-factor model with Items 20 and 24 removed, following prior evidence suggesting these items may perform inconsistently across samples (28). The two-factor and four-factor 24-item models were compared using chi-square difference tests because they were fitted to the same item set and were formally nested. The 22-item model was treated as a re-specified shortened model rather than a strictly nested model, because it removed observed indicators from the analysis; therefore, its fit was evaluated using absolute and incremental fit indices, SRMR changes, and inspection of the potentially problematic item loadings from the 24-item model.

Pearson correlation coefficients were computed to examine associations among the four factors, with coefficients of 0.10, 0.30, and 0.50 interpreted as small, medium, and large effect sizes, respectively (69). Internal consistency reliability was assessed using both Cronbach’s alpha (*α*) and McDonald’s omega (*ω*), with values ≥ 0.70 considered acceptable (70–74). McDonald’s omega was included as a complementary reliability estimate because it does not assume tau-equivalence and is considered a more appropriate measure of composite reliability for multidimensional constructs (71, 72).

#### 2.4.1 Cross-informant discrepancy analysis

To examine systematic differences between parent and child ratings, paired-samples *t*-tests were conducted for each of the four SRAS-R subscales, comparing parent and child mean scores across the 171 dyads. Effect sizes were quantified using Cohen’s *d* for paired samples, with values of 0.20, 0.50, and 0.80 interpreted as small, medium, and large effects, respectively (69).

To assess the degree of absolute agreement between parent and child reports, intraclass correlation coefficients (ICC) were computed for each subscale using a two-way mixed-effects model with absolute agreement for single measures (75–77). ICC values were interpreted following established guidelines: values below 0.40 indicate poor agreement, 0.40– 0.59 fair agreement, 0.60–0.74 good agreement, and 0.75 or above excellent agreement (77, 78). The combination of paired *t*-tests and ICC provides complementary information: *t*-tests detect systematic mean-level biases between informants, whereas ICC captures the degree of absolute consistency in ratings across the full range of scores.

## 3 Results

### 3.1 Participant characteristics

A total of 171 adolescents with depressive disorders and school refusal behavior and their caregivers participated in the study. The participant flow is illustrated in **Figure 2**, and sociodemographic characteristics are summarized in **Table 1**. The sample had a mean age of 15.5 years (*SD* = 1.90), with a preponderance of female participants (67.3%), consistent with the higher prevalence of depressive disorders among adolescent girls (27, 79). Two-thirds of participants (66.7%) were either on leave or suspended from school, reflecting the clinical severity of SRB in this sample. The majority of families reported monthly per capita income above 10,000 RMB (62.0%), and parental education levels were relatively high, suggesting that the sample represented a predominantly middle- to upper-middle-class urban population.

### 3.2 Confirmatory factor analysis

**Figure 3:**
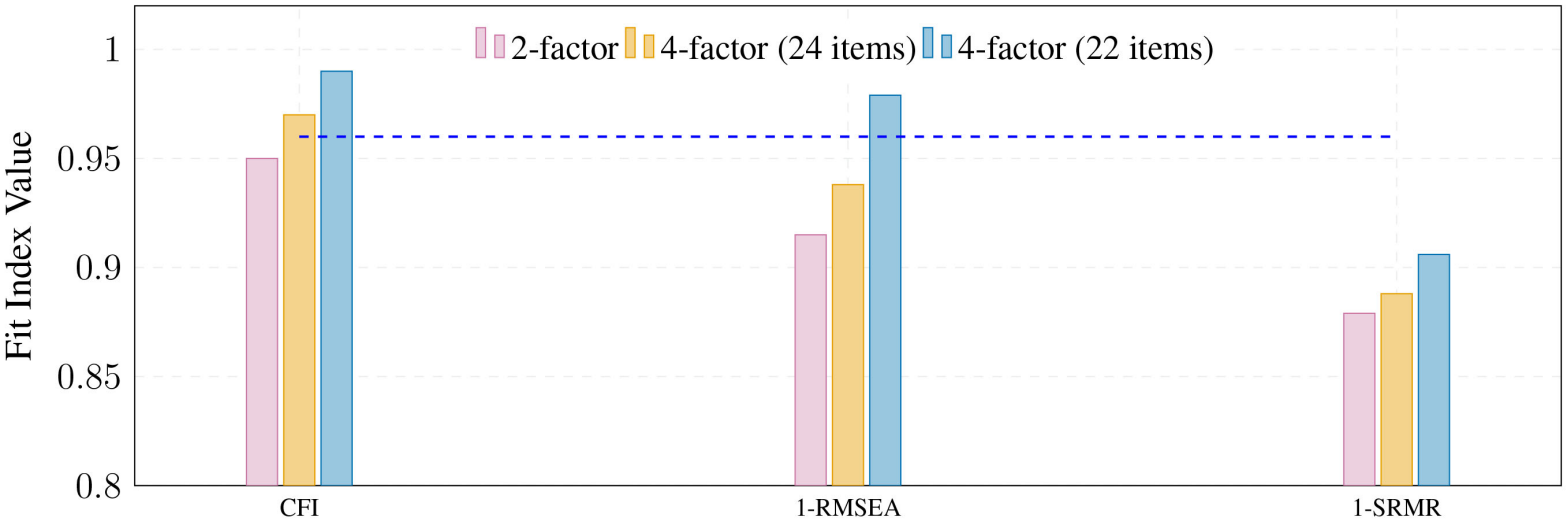
Comparison of model fit indices across the three CFA models (parent report). For visual comparability, RMSEA and SRMR values are displayed as 1−RMSEA and 1−SRMR, respectively, so that higher values indicate better fit for all indices. The dashed horizontal line indicates the recommended threshold for CFI (≥ 0.96). A color-blind-friendly palette and numeric value labels are used to support interpretation without relying on color alone.

Three competing factor models were evaluated for both parent and child reports (see **Table 2** for full fit indices and **Figure 3** for a visual comparison of fit indices across models). For the formally nested 24-item models, chi-square difference tests were used to compare the two-factor and four-factor specifications.

**Table 2:** Fit indices for confirmatory factor analysis models of the SRAS-R for parent and child reports.

Report	Model	χ2	df	p	CFI	RMSEA	SRMR
Parent	Two-factor Four-factor (24 items)	412.567 328.456	251 246	<.001 .015	0.95 0.97	0.085 0.062	0.121 0.112
	Four-factor (22 items)	221.345	203	.543	0.99	0.021	0.094
Child	Two-factor Four-factor (24 items)	438.912 351.789	251 246	<.001 .009	0.94 0.96	0.092 0.068	0.128 0.115
	Four-factor (22 items)	238.671	203	.412	0.98	0.028	0.097

CFA, confirmatory factor analysis; CFI, comparative fit index; RMSEA, root mean square error of approximation; SRMR, standardized root mean square residual. DWLS estimation was used. Recommended thresholds: CFI ≥ 0.96; RMSEA ≤ 0.08; SRMR ≤ 0.09. SRMR values for the 22-item models remained slightly above the prespecified threshold and were interpreted alongside CFI, RMSEA, and item-level results.

#### 3.2.1 Two-factor model

The two-factor model, which collapsed the four functional dimensions into negative reinforcement (Factors 1 and 2) and positive reinforcement (Factors 3 and 4), demonstrated poor fit for both parent and child reports. For parent reports, the model yielded *χ*^2^(251) = 412.567, *p <.*001, CFI = 0.95, RMSEA = 0.085, and SRMR = 0.121. For child reports, the two-factor model fit was similarly inadequate: *χ*^2^(251) = 438.912, *p <.*001, CFI = 0.94, RMSEA = 0.092, and SRMR = 0.128. All fit indices fell below recommended thresholds, with the exception of CFI for the parent report, which approached but did not reach the 0.96 criterion.

#### 3.2.2 Four-factor model with all 24 items

The initial CFA of the four-factor model with all 24 items demonstrated partial fit for both informant groups. For parent reports, the model showed acceptable CFI (0.97) and RMSEA (0.062, *p* = .321), but the chi-square test was significant (*χ*^2^(246) = 328.456, *p* = .015) and the SRMR exceeded the recommended cutoff (0.112). A similar pattern emerged for child reports: CFI = 0.96, RMSEA = 0.068 (*p* = .214), *χ*^2^(246) = 351.789 (*p* = .009), and SRMR = 0.115. Chi-square difference tests indicated that the fourfactor 24-item model fit significantly better than the two-factor 24-item model for both parent reports (Δ*χ*^2^(5) = 84.111, *p <.*001) and child reports (Δ*χ*^2^(5) = 87.123, *p <.*001). These results indicate that collapsing the four functional dimensions into two broader categories does not adequately capture the structure of SRB, consistent with findings from prior validation studies (28–30).

Inspection of the 24-item model further focused on Factor 4 because prior studies have identified Items 20 and 24 as potentially problematic. In the 24-item ordinal DWLS model, Item 20 showed a very weak and non-significant standardized loading for parent reports (*λ* = .085, *p* = .309) and a modest loading for child reports (*λ* = .429, *p <.*001). In contrast, Item 24 showed adequate standardized loadings for both parent (*λ* = .624, *p <.*001) and child reports (*λ* = .788, *p <.*001). Thus, the item-level evidence from the present sample directly supported concern about Item 20, whereas the decision to also evaluate a 22-item model excluding Item 24 was based primarily on prior SRAS-R validation literature and the goal of testing the previously recommended shortened Factor 4 specification.

#### 3.2.3 Four-factor model without items 20 and 24

**Figure 4:**
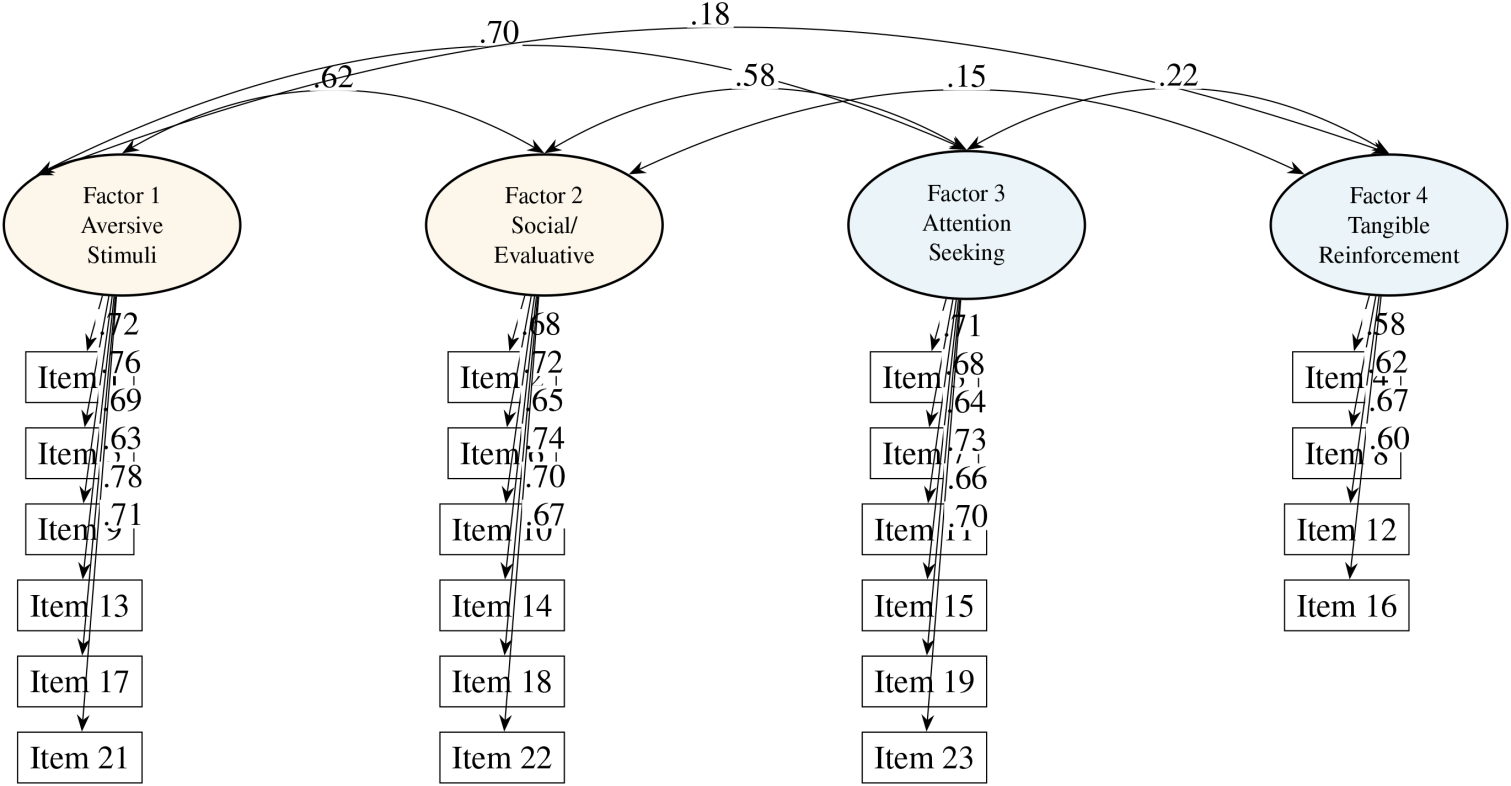
Path diagram of the four-factor confirmatory factor analysis model for the SRAS-R (parent report; Items 20 and 24 removed). Ellipses represent latent factors; rectangles represent observed items. Single-headed arrows indicate standardized factor loadings; double-headed curved arrows indicate interfactor correlations. Orange-shaded factors represent negative reinforcement dimensions; blue-shaded factors represent positive reinforcement dimensions. Numeric loading and correlation labels are provided to support interpretation in grayscale or color-blind viewing. All factor loadings are significant at *p <.*001.

Based on the poor parent loading for Item 20 and prior theoretical and empirical evidence that Items 20 and 24 may perform inconsistently in the SRAS-R (28, 32, 38), the four-factor model was re-estimated after removing both items. The revised 22-item model yielded strong CFI and RMSEA values for parent reports: *χ*^2^(203) = 221.345, *p* = .543, CFI = 0.99, RMSEA = 0.021 (*p* = .876), and SRMR = 0.094. For child reports, the model also demonstrated strong CFI and RMSEA values: *χ*^2^(203) = 238.671, *p* = .412, CFI = 0.98, RMSEA = 0.028 (*p* = .792), and SRMR = 0.097. CFI and RMSEA met recommended thresholds for both informant versions, whereas SRMR values remained slightly above the prespecified 0.09 criterion but improved substantially from the 24-item model and were interpreted as marginally acceptable in light of the DWLS estimator and the otherwise strong fit pattern. Because the 22-item model removed observed indicators, it was not treated as formally nested within the 24-item model for chi-square difference testing. Standardized factor loadings for the final model are presented in **Table 3** and visually depicted in the path diagram in **Figure 4**.

**Table 3:** Standardized factor loadings from confirmatory factor analysis and reliability of the SRAS-R (four-factor model without Items 20 and 24).

Factor/Item	Item content (abbreviated)	Parent	Child
Factor 1: Avoidance of aversive stimuli			
Item 1	How often does the child have bad feelings about school?	.72	.68
Item 5	How often does the child stay away from things at school?	.76	.71
Item 9	How often does the child feel worse at school vs. home?	.69	.65
Item 13	How often would the child rather be home-schooled?	.63	.58
Item 17	How often does the child refuse due to discomfort?	.78	.74
Item 21	How often would the child rather not be in school?	.71	.67
α/ω		.815/.820	.811/.817
Factor 2: Escape from social/evaluative situations			
Item 2	How often does the child avoid other kids at school?	.68	.62
Item 6	How often does the child avoid speaking to others?	.72	.66
Item 10	How often does the child feel embarrassed at school?	.65	.58
Item 14	How often does the child refuse due to social fears?	.74	.70
Item 18	How often does the child feel nervous around others?	.70	.64
Item 22	How often does the child refuse due to evaluative fears?	.67	.61
α/ω		.784/.790	.700/.710
Factor 3: Pursuit of attention from significant others			
Item 3	How often does the child try to get parent attention?	.71	.76
Item 7	How often does the child want parent nearby?	.68	.73
Item 11	How often does the child want to talk to parents?	.64	.70
Item 15	How often does the child prefer to be with family?	.73	.78
Item 19	How often does the child want parent to stay home?	.66	.72
Item 23	How often does the child refuse to get parent attention?	.70	.75
α/ω		.783/.788	.864/.868
Factor 4: Pursuit of tangible reinforcement			
Item 4	How often would the child enjoy activities outside school?	.58	.55
Item 8	How often does the child think about fun outside school?	.62	.59
Item 12	How often does the child refuse to pursue activities?	.67	.63
Item 16	How often would the child have more fun outside school?	.60	.57
α/ω		.664/.670	.716/.722

All factor loadings were significant at *p <.*001. Items 20 and 24 were removed in the final 22-item model based on prior SRAS-R validation literature, global model fit, and item-level inspection of the 24-item model. *α* = Cronbach’s alpha; *ω* = McDonald’s omega. Factor loadings are from the four-factor CFA model estimated using DWLS.

All standardized factor loadings were statistically significant (*p <.*001) and exceeded 0.50, ranging from 0.58 to 0.78 for parent reports and from 0.55 to 0.78 for child reports. For parent reports, Factor 1 loadings ranged from 0.63 (Item 13) to 0.78 (Item 17); Factor 2 loadings ranged from 0.65 (Item 10) to 0.74 (Item 14); Factor 3 loadings ranged from 0.64 (Item 11) to 0.73 (Item 15); and Factor 4 loadings ranged from 0.58 (Item 4) to 0.67 (Item 12). A similar pattern was observed for child reports, with Factor 3 exhibiting the highest maximum loading (0.78 for Items 15).

### 3.3 Descriptive statistics for SRAS-R subscales

**Figure 5:**
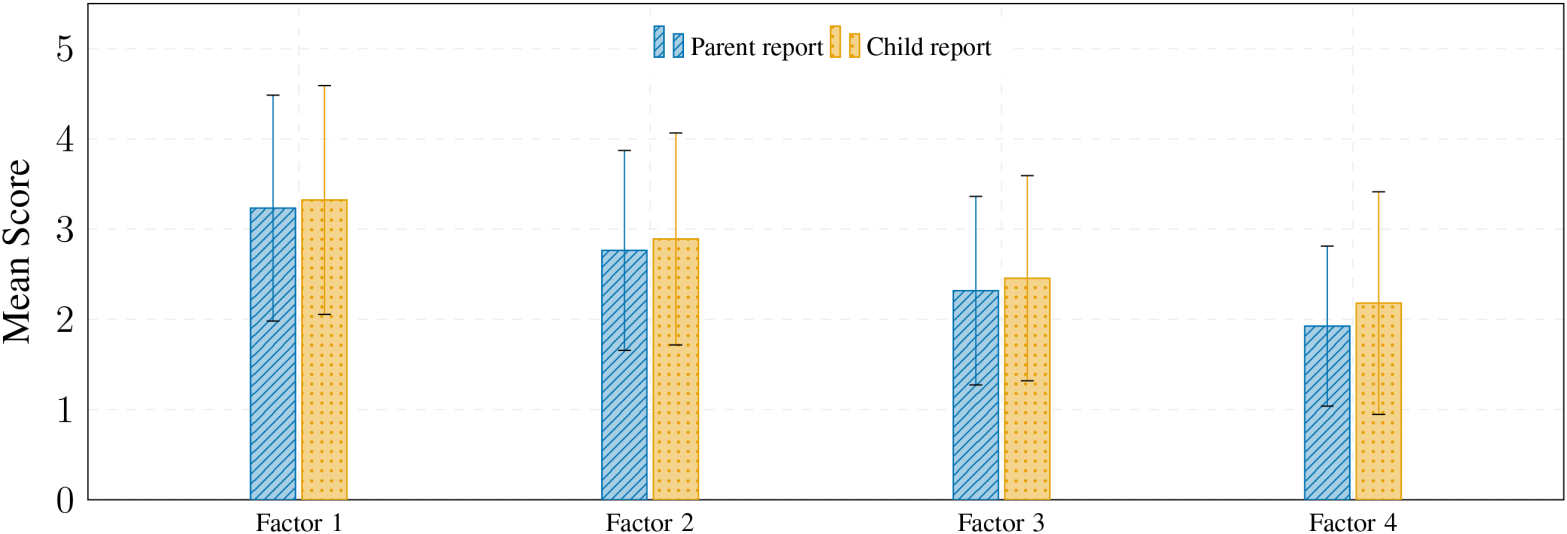
Mean scores on SRAS-R subscales for parent and child reports (*N* = 171). Error bars represent standard deviations. Factor 1 = Avoidance of aversive stimuli; Factor 2 = Escape from social/evaluative situations; Factor 3 = Pursuit of attention from significant others; Factor 4 = Pursuit of tangible reinforcement outside school. Both informants show the same rank ordering of factors, with Factor 1 most endorsed and Factor 4 least endorsed. Distinct patterns, numeric labels, and a blue/orange palette are used to improve accessibility for grayscale and color-blind readers.

Descriptive statistics for the four SRAS-R subscales are presented in **Table 4** and illustrated in **Figure 5**. For parent reports, Factor 1 (avoidance of aversive school situations) yielded the highest mean score (*M* = 3.233, *SD* = 1.252), followed by Factor 2 (escape from social/evaluative situations; *M* = 2.764, *SD* = 1.108), Factor 3 (seeking attention; *M* = 2.318, *SD* = 1.045), and Factor 4 (seeking tangible reinforcement; *M* = 1.925, *SD* = 0.887). A similar rank ordering emerged for child reports: Factor 1 (*M* = 3.323, *SD* = 1.268), Factor 2 (*M* = 2.891, *SD* = 1.175), Factor 3 (*M* = 2.456, *SD* = 1.137), and Factor 4 (*M* = 2.180, *SD* = 1.234). Both parent and child reports identified avoidance of aversive stimuli as the primary functional motivation and pursuit of tangible reinforcement as the least prominent (H5 confirmed).

**Table 4:** Descriptive statistics for SRAS-R subscale scores by informant (*N* = 171).

Subscale	Parent report		Child report	
	M	SD	M	SD
Factor 1: Avoidance of aversive stimuli	3.233	1.252	3.323	1.268
Factor 2: Escape from social/evaluative	2.764	1.108	2.891	1.175
Factor 3: Pursuit of attention	2.318	1.045	2.456	1.137
Factor 4: Pursuit of tangible reinforcement	1.925	0.887	2.180	1.234

Scores range from 0 (never) to 6 (always). Higher scores indicate greater endorsement of the respective function. Factor scores are computed as the mean of constituent items (6 items for Factors 1–3; 4 items for Factor 4 after removal of Items 20 and 24).

### 3.4 Inter-factor correlations

**Figure 6:**
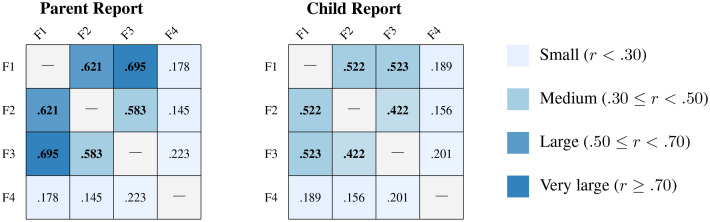
Heatmap visualization of inter-factor correlations for parent report (left) and child report (right). F1 = avoidance of aversive stimuli; F2 = escape from social/evaluative situations; F3 = pursuit of attention; F4 = pursuit of tangible reinforcement. Bold values indicate *p <.*001. Darker blue shading indicates stronger correlations, and all cells include numeric correlation values so the figure remains interpretable in grayscale or color-blind viewing. Factor 4 shows consistently weak correlations with Factors 1–3 across both informants, supporting the theoretical distinctiveness of positively reinforced school refusal.

Inter-factor correlations are presented in **Table 5** and visualized in **Figure 6**. For parent reports, Factor 1 was strongly and positively correlated with Factor 3 (*r* = .695, *p <.*001) and Factor 2 (*r* = .621, *p <.*001). Factor 2 and Factor 3 showed a moderate-to-strong positive correlation (*r* = .583, *p <.*001). Correlations between Factor 4 and the remaining factors were uniformly small (*r*s = .155–.223, *p*s *<.*05). For child reports, Factor 1 correlated strongly with Factor 2 (*r* = .522, *p <.*001) and Factor 3 (*r* = .523, *p <.*001), while Factor 2 and Factor 3 showed a moderate correlation (*r* = .422, *p <.*001). Factor 4 again demonstrated the weakest associations with other factors (*r*s = .145–.189, *p*s *<.*05), supporting the theoretical distinctiveness of the tangible reinforcement dimension (H4 partially confirmed).

**Table 5:** Pearson correlations among SRAS-R factors for parent (below diagonal) and child (above diagonal) reports.

Factor	Factor 1	Factor 2	Factor 3	Factor 4
Factor 1: Avoidance of aversive stimuli	—	.522∗∗∗	.523∗∗∗	.189∗
Factor 2: Escape from social/evaluative	.621∗∗∗	—	.422∗∗∗	.156∗
Factor 3: Pursuit of attention	.695∗∗∗	.583∗∗∗	—	.201∗
Factor 4: Pursuit of tangible reinforcement	.178∗	.145∗	.223∗	—

Parent report correlations are below the diagonal; child report correlations are above the diagonal. *N* = 171 for both informants.

^∗^ *p <.*05. ^∗∗∗^*p <.*001.

### 3.5 Internal consistency reliability

Internal consistency estimates for the SRAS-R subscales are presented in **Table 3**. For parent reports, Cronbach’s alpha values ranged from 0.664 (Factor 4) to 0.815 (Factor 1). McDonald’s omega values were comparable, ranging from 0.670 (Factor 4) to 0.820 (Factor 1). For child reports, Cronbach’s alpha ranged from 0.700 (Factor 2) to 0.864 (Factor 3), with McDonald’s omega values ranging from 0.710 (Factor 2) to 0.868 (Factor 3). The majority of coefficients met or exceeded the 0.70 threshold (70); however, Factor 4 in the parent version fell below this conventional criterion (*α* = .664, *ω* = .670), indicating only marginal internal consistency for parent-reported tangible reinforcement. Thus, H3 was largely but not fully supported. The close correspondence between *α* and *ω* values across all subscales suggests approximate tau-equivalence of items within each factor, supporting the appropriateness of the congeneric measurement model (71, 73).

### 3.6 Cross-informant discrepancy

Results of the paired-samples *t*-tests and intraclass correlation coefficients (ICC) examining parent–child discrepancies across the four SRAS-R subscales are presented in **Table 6**.

**Table 6:** Cross-informant discrepancy: paired-samples *t*-tests and intraclass correlation coefficients for SRAS-R subscales (*N* = 171 dyads).

Subscale	Parent		Child		t(170)	p	d	ICC	95% CI
	M	SD	M	SD					
Factor 1: Avoidance of aversive stimuli	3.233	1.252	3.323	1.268	−1.58	.116	0.12	.62	[.52,.70]
Factor 2: Escape from social/evaluative	2.764	1.108	2.891	1.175	−2.41	.017	0.18	.51	[.40,.61]
Factor 3: Pursuit of attention	2.318	1.045	2.456	1.137	−1.89	.061	0.14	.60	[.50,.69]
Factor 4: Pursuit of tangible reinforcement	1.925	0.887	2.180	1.234	−3.52	<.001	0.27	.45	[.33,.56]

Paired-samples *t*-tests compared parent and child mean scores for each subscale. Negative *t*-values indicate that children reported higher scores than parents. *d* = Cohen’s *d* for paired samples. ICC = intraclass correlation coefficient (two-way mixed-effects model, absolute agreement, single measures). CI = confidence interval.

#### 3.6.1 Paired-samples *t*-Tests

Paired-samples *t*-tests revealed that children reported significantly higher scores than parents on Factor 2 (escape from social/evaluative situations; *t*(170) = −2.41, *p* = .017, *d* = 0.18) and Factor 4 (pursuit of tangible reinforcement; *t*(170) = −3.52, *p <.*001, *d* = 0.27). The differences for Factor 1 (avoidance of aversive stimuli; *t*(170) = −1.58, *p* = .116, *d* = 0.12) and Factor 3 (pursuit of attention; *t*(170) = −1.89, *p* = .061, *d* = 0.14) did not reach statistical significance, although a trend was observed for Factor 3. Overall, the pattern indicated that children consistently rated all four functional motivations somewhat higher than their parents, with the most pronounced discrepancies in the internally driven and externally oriented dimensions (Factors 2 and 4). Effect sizes were small across all comparisons (H6 partially confirmed).

#### 3.6.2 Intraclass correlation coefficients

ICC analyses using the two-way mixed-effects model (absolute agreement, single measures) indicated fair to good agreement between parent and child reports across the four subscales. Factor 1 showed the highest ICC (ICC = 0.62, 95% CI [0.52, 0.70]), indicating good agreement. Factor 3 also demonstrated good agreement (ICC = 0.60, 95% CI [0.50, 0.69]). Factor 2 (ICC = 0.51, 95% CI [0.40, 0.61]) and Factor 4 (ICC = 0.45, 95% CI [0.33, 0.56]) showed fair agreement. The lower ICC for Factor 2 is noteworthy given that this dimension captures internal social and evaluative anxieties that may be less observable to parents, while the lower ICC for Factor 4 may reflect differential awareness of tangible reinforcers between parents and adolescents.

## 4 Discussion

The present study examined the psychometric properties of the Chinese version of the SRAS-R in a clinical sample of 171 adolescents with depressive disorders who exhibited school refusal behavior. To our knowledge, this is the first study to evaluate the SRAS-R in a Chinese clinical population with diagnosed psychiatric disorders. The findings support the four-factor structure of the instrument after removing Items 20 and 24, indicate marginal to excellent internal consistency across subscales, and show meaningful parent–child discrepancies. These results provide initial validity evidence for using the Chinese SRAS-R in this clinical context, while also identifying boundaries that require further study.

### 4.1 Factorial validity and model comparison

Consistent with our hypotheses and prior cross-cultural validation studies (2, 28–32, 38), the four-factor model demonstrated better fit than the two-factor model. The chi-square difference tests supported retaining the four distinct functional dimensions rather than collapsing them into broader negative and positive reinforcement categories. This finding aligns with the theoretical model proposed by Kearney and Silverman (16) and Kearney (2), which emphasizes the importance of distinguishing among four functional motivations for school refusal, and is consistent with the growing evidence base from international validation studies (29–32).

The initial four-factor model with all 24 items showed partial fit, with CFI and RMSEA values meeting thresholds but SRMR values and chi-square tests indicating some misfit. Item-level inspection indicated that Item 20 performed poorly for parent reports, whereas Item 24 did not show a weak loading in the present sample. This pattern only partially replicates prior findings by Kearney (28), who reported that Items 20 and 24 loaded poorly on Factor 4 in the original English version. Item 20 asks whether going to school would be easier if the child could do more preferred activities after school, and its weak parent loading suggests that parents may not view after-school reinforcement as directly related to current refusal behavior. Item 24 assesses whether the child would rather find enjoyable things to do outside school; its adequate loading in the present sample suggests that it may still capture meaningful tangible reinforcement for some Chinese adolescents. Therefore, the 22-item model should be interpreted as a literature-informed sensitivity model rather than definitive evidence that both items are invalid in this sample. In a Chinese clinical context, these items may also be interpreted through expectations about academic obligation and parental monitoring: spending time with friends or engaging in leisure activities during school hours may be viewed as broadly undesirable or rule-breaking, regardless of whether these activities actually reinforce school refusal. Future work should test whether revised items can better capture culturally specific out-of-school reinforcers such as online activities, rest, private tutoring avoidance, or peer contact outside formal school settings.

The 22-item model showed strong CFI and RMSEA values, but the SRMR values remained slightly above the prespecified cutoff. This pattern suggests that the shortened four-factor structure is promising but should be interpreted cautiously rather than as uniformly excellent across all indices. The finding is consistent with prior work suggesting difficulties with the original Factor 4 item set, while also underscoring the need for replication in independent Chinese clinical samples.

### 4.2 Inter-factor correlations and theoretical implications

The pattern of inter-factor correlations was largely consistent with theoretical expectations and prior empirical findings. The two negative reinforcement factors (Factor 1: avoidance of aversive situations; Factor 2: escape from social/evaluative situations) were moderately to strongly correlated for both parent (*r* = .621) and child (*r* = .522) reports, suggesting that youth who avoid school due to general negative affectivity also tend to experience social-evaluative anxiety (80, 81). Similarly, Factor 1 showed strong correlations with Factor 3 (seeking attention) for parent reports (*r* = .695), indicating that in this clinical sample, avoidance behaviors and attention-seeking behaviors frequently co-occur, possibly reflecting the complex clinical presentations typical of depressed adolescents with school refusal (13, 17, 19).

Factor 4 (seeking tangible reinforcement) consistently showed the weakest correlations with other factors across both informant groups (*r*s = .145–.223), supporting the conceptual distinctiveness of this positive reinforcement dimension. This pattern mirrors findings from Spanish (30, 31), German (32), Norwegian (38), and American (29) samples, providing robust cross-cultural evidence for the relative independence of the tangible reinforcement function. This is consistent with the theoretical model, which posits that tangible reinforcement represents a functionally distinct motivation that may operate relatively independently of the anxiety-driven factors and is more characteristic of truancy-related presentations (2, 5, 13).

An unexpected finding was the strong correlation between Factor 1 and Factor 3 in parent reports (*r* = .695), which was the highest inter-factor correlation observed. This may reflect a pattern specific to depressed adolescents, in whom negative affectivity and increased dependence on caregivers frequently co-occur (18, 19). Clinically, this suggests that interventions for depressed school-refusing youth may need to simultaneously address school-related avoidance and family interaction patterns (1, 82).

The reliability results further suggest that Factor 4 should be interpreted with caution in parent reports. Although the child-reported Factor 4 reliability was acceptable, the parent-reported alpha and omega were both below.70. This may reflect the shortened four-item composition after removing Items 20 and 24, limited parental awareness of tangible reinforcers outside school, or heterogeneity in what constitutes “tangible reinforcement” for depressed adolescents in contemporary Chinese settings. Clinically, low parent-reported Factor 4 scores should therefore not be interpreted as definitive evidence that tangible reinforcement is absent; they should be integrated with adolescent self-report, clinical interview, and contextual information about school absence.

### 4.3 Predominance of factor 1 in depressed adolescents

A notable finding was that Factor 1 (avoidance of school-related stimuli provoking negative affectivity) yielded the highest mean scores for both parent (*M* = 3.233) and child (*M* = 3.323) reports, confirming Hypothesis 5. This finding is clinically meaningful given that the sample comprised exclusively adolescents with depressive disorders, a population characterized by pervasive negative affect, anhedonia, and heightened sensitivity to aversive stimuli (8, 18, 79). The prominence of negative affectivity-driven avoidance in this sample suggests that interventions targeting school refusal in depressed adolescents should prioritize addressing negative emotional responses to the school environment, such as through cognitive-behavioral strategies for emotion regulation, behavioral activation, and gradual exposure to school settings (1, 49, 52, 83).

In contrast, Factor 4 (seeking tangible reinforcement) obtained the lowest mean scores for parent reports (*M* = 1.925), suggesting that positive reinforcement contingencies outside of school play a comparatively minor role in maintaining school refusal among depressed adolescents. This finding aligns with the clinical characterization of school refusal as primarily anxiety- and depression-driven rather than truancy-driven in clinical populations (13, 17, 19, 84). Previous research has similarly noted that negatively reinforced school refusal tends to predominate in clinical samples (2, 29, 47), whereas positively reinforced school refusal is more characteristic of truancy-related presentations (5, 85, 86).

The hierarchical pattern of factor means (Factor 1 *>* Factor 2 *>* Factor 3 *>* Factor 4) observed in both parent and child reports is consistent with the profile reported by Gonzálvez et al. (47) in a community sample and by Walter et al. (32) in a clinical sample, suggesting that this rank ordering may be relatively stable across cultural contexts and sample types when SRB is primarily driven by emotional distress.

### 4.4 Cross-informant discrepancy and parent–child agreement

The cross-informant discrepancy analyses provided nuanced insights into the agreement and divergence between parent and child perspectives on school refusal functions (H6 partially confirmed). While both informants identified Factor 1 as the primary functional motivation and Factor 4 as the least prominent—and the rank ordering of subscale means was identical across informants—paired-samples *t*-tests revealed statistically significant discrepancies for Factors 2 and 4.

The finding that children reported significantly higher scores than parents on Factor 2 (escape from social/evaluative situations; *p* = .017, *d* = 0.18) suggests that parents may underestimate the social and evaluative anxiety experienced by their depressed adolescents. This is clinically significant because it implies that relying solely on parent reports may lead to an underappreciation of the social-evaluative component of school refusal in this population. This finding aligns with the broader multi-informant literature demonstrating that internalizing experiences are less accessible to external observers (54, 55). Clinically, this discrepancy highlights the importance of directly assessing adolescents’ own perceptions of social and evaluative threats at school, as these internal motivations may be underrepresented in parental accounts.

Similarly, children reported significantly higher scores on Factor 4 (pursuit of tangible reinforcement; *p <.*001, *d* = 0.27), indicating that parents may be less aware of the tangible reinforcers available outside school that contribute to their child’s school refusal. Adolescents may be more cognizant of or willing to endorse enjoyable activities and social opportunities that compete with school attendance (2).

The ICC analyses revealed fair to good agreement across factors, with Factor 1 (ICC = 0.62) and Factor 3 (ICC = 0.60) showing good agreement, and Factors 2 (ICC = 0.51) and 4 (ICC = 0.45) showing only fair agreement. The pattern of ICC values is consistent with the *t*-test findings: factors for which significant mean-level discrepancies were observed also showed lower absolute agreement. The relatively higher ICC for Factor 1 may reflect the fact that negative affectivity and distress-driven avoidance produce observable behavioral manifestations (e.g., somatic complaints, crying, resistance to leaving home) that are accessible to both informants (1). In contrast, the lower ICC for Factor 2 underscores that social-evaluative anxiety is a more private, internally experienced phenomenon that parents may not readily observe (54).

These findings have clear clinical implications: in assessments of school-refusing depressed adolescents, clinicians must directly inquire about the adolescent’s own experiences of social anxiety and evaluative fear rather than relying primarily on parental reports. The systematic underestimation by parents on these dimensions reinforces the necessity of administering both parent and child versions of the SRAS-R to obtain a comprehensive functional profile (1, 13, 87). This point may be especially relevant in Chinese families, where adolescents may not spontaneously disclose school-related shame, peer conflict, or evaluative fears to parents, and parents may interpret absence primarily through academic responsibility or observable distress. Discrepancies between informant reports should therefore not be dismissed as measurement noise but treated as clinically informative signals that can guide family-based interventions and identify areas where parent psychoeducation may be warranted (55, 56).

### 4.5 Additional validity evidence

Although the strongest evidence in this study comes from internal structure and reliability, several findings provide additional support for the validity of the Chinese SRAS-R. First, the translation and cultural adaptation process included forward translation, back-translation, expert review, and pilot testing with adolescents and parents, providing preliminary response-process evidence that the translated items were understandable and culturally appropriate. Second, the pattern of inter-factor correlations was theoretically coherent: negative reinforcement factors were more strongly associated with one another than with tangible reinforcement, and Factor 4 remained relatively distinct across informants. Third, the clinical profile of subscale means was consistent with expectations for adolescents with depressive disorders, with Factor 1 emerging as the most endorsed function and tangible reinforcement the least endorsed function. Fourth, parent–child agreement analyses provided evidence based on informant relationships, showing stronger agreement for more observable forms of distress and weaker agreement for social/evaluative anxiety and tangible reinforcement.

At the same time, this validity evidence remains incomplete. The study did not include external criterion measures such as objective school attendance records, validated anxiety and depression scales, teacher reports, clinician-rated impairment, or treatment outcomes. Therefore, the present findings support the use of the Chinese SRAS-R as a structured functional assessment tool, but they do not yet establish its full convergent, discriminant, criterion-related, or predictive validity. Future research should explicitly test these forms of validity evidence before the scale is used for high-stakes diagnostic or placement decisions.

### 4.6 Clinical implications

The present findings have several important clinical implications for the assessment and treatment of school refusal in Chinese adolescents with depressive disorders:

First, the Chinese SRAS-R offers clinicians working with Chinese adolescents a structured, theory-based tool for assessing the functional motivations underlying SRB, although further external validity evidence is still needed. This is particularly important given that effective interventions for school refusal are function-specific (1, 13): youth whose refusal is maintained by negative affectivity avoidance may benefit most from relaxation training, cognitive restructuring, and systematic desensitization, whereas those driven by attention-seeking may respond better to parent training in contingency management (1, 82, 83).

Second, the finding that avoidance of aversive school situations is the predominant function in depressed adolescents can guide clinicians in prioritizing treatment targets. Cognitive-behavioral interventions that combine exposure-based approaches with cognitive restructuring of negative school-related cognitions and behavioral activation strategies may be particularly effective for this population (49, 50, 52, 82, 83). The integration of depression-specific treatment components (e.g., behavioral activation, positive activity scheduling) with school refusal interventions represents a promising direction for clinical practice (51).

Third, the relatively lower scores on Factor 4 suggest that clinicians working with depressed, schoolrefusing adolescents may need to focus less on external contingency management and more on addressing the internal emotional barriers to school attendance. However, the non-trivial child-reported scores on Factor 4 suggest that tangible reinforcers should not be entirely overlooked in comprehensive assessment.

Fourth, the similar patterns observed across parent and child reports support the clinical utility of multiinformant assessment and suggest that both perspectives provide valuable and complementary information for treatment planning. Discrepancies between parent and child reports, when they occur, can themselves serve as clinically informative data points that guide family-based interventions (1, 55).

### 4.7 Limitations and future directions

Several limitations should be considered when interpreting these findings. First, the study did not include external criterion measures, which is a substantial limitation for a clinical validation study. Although the findings provide evidence based on internal structure, reliability, response processes, and theoretically coherent informant patterns, they do not establish convergent, discriminant, criterion-related, or predictive validity. Future studies should examine associations between SRAS-R subscales and objective school attendance records, validated anxiety measures (88, 89), depression symptom measures (90–92), clinician-rated impairment, family functioning, teacher reports, and treatment outcomes.

Second, the sample was drawn from a single clinical site in Beijing and was characterized by relatively high socioeconomic status and high parental education. The findings therefore should not be generalized broadly to all Chinese adolescents with SRB, rural or lower-SES populations, community samples, or healthcare settings with different referral pathways. Future studies should examine the SRAS-R’s properties across diverse geographic regions, school systems, socioeconomic backgrounds, and clinical services.

Third, all participants had depressive disorders, and adolescents with ADHD and other neurodevelopmental conditions were excluded. This design strengthened clinical specificity but limits applicability to populations in which school refusal is maintained by attentional difficulties, executive dysfunction, impulsivity, bullying vulnerability, or autism-related social demands (38, 57, 58). Fourth, the predominance of female participants (67.3%) may have influenced the observed functional profile, particularly the prominence of negative affectivity and social-evaluative concerns. Larger and more balanced samples are needed to test measurement invariance and latent mean differences across gender, age, and diagnostic subgroups.

Fifth, the cross-sectional design precludes conclusions about the temporal stability of the factor structure; test–retest reliability should be examined in future research to establish the stability of SRAS-R scores over time. Sixth, the sample size of 171, while adequate for the CFA models tested given the DWLS estimation method (64, 65), is relatively modest and may limit the detection of small effects or the stability of parameter estimates. Seventh, the study relied on self- and parent-report data, which are subject to response biases; future studies could incorporate teacher reports, clinician ratings, ecological momentary assessment, or objective attendance data to triangulate findings. Finally, Items 20 and 24 were removed based on prior theoretical and empirical grounds (28), and the improved fit of the shortened model supports further investigation of a 22-item scoring approach; however, Item 24 showed adequate loadings in the present sample, so its removal should be interpreted cautiously. Future research should examine whether revised or culturally adapted versions of these items could improve the measurement of Factor 4 in Chinese populations.

### 4.8 Conclusion

The present study provides initial evidence for the psychometric properties of the Chinese SRAS-R in a single-site clinical sample of adolescents with depressive disorders and school refusal behavior. The four-factor model with Items 20 and 24 removed showed strong CFI and RMSEA values for both parent and child reports, although SRMR remained marginal and parent-reported Factor 4 reliability was below the conventional threshold. The pattern of subscale means, with Factor 1 as the most endorsed function, is consistent with the depression-related negative affectivity characteristic of this clinical population. Cross-informant discrepancy analyses revealed that parents reported lower social/evaluative anxiety and tangible reinforcement motivations than adolescents, with ICC values indicating only fair to good absolute agreement. These findings support the Chinese SRAS-R as a promising functional assessment tool for depressed adolescents with SRB, but conclusions should remain limited to preliminary internal-structure and reliability evidence until convergent, discriminant, criterion-related, predictive, and measurementinvariance evidence is available in more diverse samples.

## Data Availability

The original contributions presented in the study are included in the article/supplementary material. Further inquiries can be directed to the corresponding author.

## References

[1] Kearney CA . School Refusal Behavior in Youth: A Functional Approach to Assessment and Treatment. Washington, DC: American Psychological Association (2001). doi: 10.1037/10426-000

[2] Kearney CA . Identifying the function of school refusal behavior: a revision of the School Refusal Assessment Scale. J Psychopathol Behav Assess. (2002) 24:235–45. doi: 10.1023/A:1020774932043 41886696

[3] Berg I . School refusal and truancy. Arch Dis Childhood. (1997) 76:90–1. doi: 10.1136/adc.76.2.90 9068294 PMC1717084

[4] Heyne D Gren-Landell M Melvin G Gentle-Genitty C . Differentiation between school attendance problems: why and how? Cogn Behav Pract. (2019) 26:8–34. doi: 10.1016/j.cbpra.2018.03.006 38826717

[5] Kearney CA . School absenteeism and school refusal behavior in youth: a contemporary review. Clin Psychol Rev. (2008) 28:451–71. doi: 10.1016/j.cpr.2007.07.012 17720288

[6] Yu Y Chen JH Lau JT Wu AM Du M Chen Y . Validation of the School Refusal Assessment Scale–Revised (SRAS-R) in the general adolescent population in China. School. Ment Health. (2024) 16:436–46. doi: 10.1007/s12310-024-09640-8 30311153

[7] Kearney CA Graczyk PA . A multidimensional, multi-tiered system of supports model to promote school attendance and address school absenteeism. Clin Child Family Psychol Rev. (2020) 23:316–37. doi: 10.1007/s10567-020-00317-1 32274598

[8] Finning K Ukoumunne OC Ford T Danielsson-Waters E Shaw L De Jager IR . The association between child and adolescent depression and poor attendance at school: a systematic review and meta-analysis. J Affect Disord. (2019) 245:928–38. doi: 10.1016/j.jad.2018.11.055 30699878

[9] Fremont WP . School refusal in children and adolescents. Am Family Phys. (2003) 68:1555–61. 14596443

[10] Flakierska-Praquin N Lindström M Gillberg C . School phobia with separation anxiety disorder: a comparative 20- to 29-year follow-up study of 35 school refusers. Compr Psychiatry. (1997) 38:17–22. doi: 10.1016/S0010-440X(97)90048-1 8980867

[11] Fleming M Fitton CA Steiner MFC McLay JS Clark D King A . Educational and health outcomes of children treated for attention-deficit/hyperactivity disorder. JAMA Pediatr. (2017) 171:e170691. doi: 10.1001/jamapediatrics.2017.0691 28459927 PMC6583483

[12] Kearney CA . Case study of the assessment and treatment of a youth with multifunction school refusal behavior. Clin Case Stud. (2002) 1:67–80. doi: 10.1177/1534650102001001005

[13] Kearney CA Albano AM . The functional profiles of school refusal behavior: diagnostic aspects. Behav Mod. (2004) 28:147–61. doi: 10.1177/0145445503259263 14710711

[14] Kearney CA Silverman WK . The evolution and reconciliation of taxonomic strategies for school refusal behavior. Clin Psy.: Sci Pract. (1996) 3:339–54. doi: 10.1111/j.1468-2850.1996.tb00087.x 40046247

[15] Elliott JG . Practitioner review: school refusal: issues of conceptualisation, assessment, and treatment. J Child Psychol Psychiatry. (1999) 40:1001–12. doi: 10.1111/1469-7610.00519 10576531

[16] Kearney CA Silverman WK . Measuring the function of school refusal behavior: the School Refusal Assessment Scale. J Clin Child Psychol. (1993) 22:85–96. doi: 10.1207/s15374424jccp2201_9 42277559

[17] Egger HL Costello EJ Angold A . School refusal and psychiatric disorders: a community study. J Am Acad Child Adolesc Psychiatry. (2003) 42:797–807. doi: 10.1097/01.CHI.0000046865.56865.79 12819439

[18] Kearney CA . Depression and school refusal behavior: a review with comments on classification and treatment. J School. Psychol. (1993) 31:267–79. doi: 10.1016/0022-4405(93)90012-8

[19] Bernstein GA Borchardt CM . School refusal: family constellation and family functioning. J Anxiety Disord. (1996) 10:1–19. doi: 10.1016/0887-6185(95)00031-3

[20] King NJ Bernstein GA . School refusal in children and adolescents: a review of the past 10 years. J Am Acad Child Adolesc Psychiatry. (2001) 40:197–205. doi: 10.1097/00004583-200102000-00014 11211368

[21] Beaton DE Bombardier C Guillemin F Ferraz MB . Guidelines for the process of cross-cultural adaptation of self-report measures. Spine. (2000) 25:3186–91. doi: 10.1097/00007632-200012150-00014 11124735

[22] Hambleton RK Merenda PF Spielberger CD . Adapting Educational and Psychological Tests for Cross-Cultural Assessment. Mahwah, NJ: Lawrence Erlbaum Associates (2005).

[23] Wild D Grove A Martin M Eremenco S McElroy S Verjee-Lorenz A . Principles of good practice for the translation and cultural adaptation process for patient-reported outcomes (PRO) measures: report of the ISPOR Task Force for translation and cultural adaptation. Value. Health. (2005) 8:94–104. doi: 10.1111/j.1524-4733.2005.04054.x 15804318

[24] Chen X Wang L Li D . Adolescents’ perceptions of parenting styles and school adjustment in China. Youth Soc. (2008) 40:432–51.

[25] Liu X Kurita H Sun Z Wang F . Risk factors for psychopathology among Chinese children. Psychiatry Clin Neurosci. (2011) 53:497–503. doi: 10.1046/j.1440-1819.1999.00579.x 10498232

[26] Sun R Gai X . School refusal behavior among Chinese adolescents: prevalence and associated factors. School. Psychol Int. (2023) 44:180–98.

[27] Tang X Tang S Ren Z Wong DFK . Prevalence of depressive symptoms among adolescents in secondary school in mainland China: a systematic review and meta-analysis. J Affect Disord. (2019) 245:498–507. doi: 10.1016/j.jad.2018.11.043 30439677

[28] Kearney CA . Confirmatory factor analysis of the School Refusal Assessment Scale–Revised: child and parent versions. J Psychopathol Behav Assess. (2006) 28:139–44. doi: 10.1007/s10862-005-9005-6 30311153

[29] Haight C Kearney CA Hendron M Schafer R . Confirmatory analyses of the School Refusal Assessment Scale–Revised: replication and extension to a truancy sample. J Psychopathol Behav Assess. (2011) 33:196–204. doi: 10.1007/s10862-011-9218-9 30311153

[30] Gonzálvez C Inglés CJ Kearney CA Vicent M Sanmartín R García-Fernández JM . School Refusal Assessment Scale–Revised: factorial invariance and latent means differences across gender and age in Spanish children. Front Psychol. (2016) 7:2011. doi: 10.3389/fpsyg.2016.02011 28082938 PMC5183572

[31] Gonzálvez C Kearney CA Lagos-San Martín N Sanmartín R Vicent M Inglés CJ . School Refusal Assessment Scale–Revised Chilean Version: factorial invariance and latent means differences across gender and age. J Psy. Assess. (2018) 36:835–43. doi: 10.1177/0734282917712173 PMC5183572 28082938

[32] Walter D von Bialy J von Wirth E Doepfner M . Psychometric properties of the german school refusal assessment scale–revised. J Psy. Assess. (2018) 36:644–8. doi: 10.1177/0734282916689641

[33] Holden B Sållman J-I . Skolenekting: Årsaker, Kartlegging Og Behandling. Norway: Kommuneforlaget (2010).

[34] Seçer İ . Adaptation of School Refusal Assessment Scale into Turkish: reliability and validity studies. Pakistan J Stat. (2014) 30:1197–202.

[35] Brandibas G Jeunier B Clanet C Fouraste R . Truancy, school refusal and anxiety. School. Psychol Int. (2004) 25:117–26. doi: 10.1177/0143034304036299

[36] Heyne DA Vreeke LJ Maric M Boelens H Van Widenfelt BM . Functional assessment of school attendance problems: an adapted version of the School Refusal Assessment Scale– Revised. J Emotional. Behav Disord. (2017) 25:178–92. doi: 10.1177/1063426616661701

[37] Maeda N Hatada S Sonoda J Takayama I . School-based intensive exposure therapy for school refusal behavior. Clin Case Stud. (2012) 11:299–311. doi: 10.1177/1534650112457456

[38] Orm S Holgersen ÅK Orm CB Daniel Á Fossum IN Løkke JA . Confirming the validity of the School-Refusal Assessment Scale—Revised in a sample of children with attentiondeficit/hyperactivity disorder. Front Psychol. (2022) 13:849303. doi: 10.3389/fpsyg.2022.849303 35345632 PMC8957079

[39] Adams D McLucas R Mitchelson H Simpson K Dargue N . Form, function and feedback on the School Refusal Assessment Scale–Revised in children on the autism spectrum. J Autism Dev Disord. (2021) 8:436–53. doi: 10.1007/s10803-021-05107-4 34081301

[40] Munkhaugen EK Torske T Gjevik E Nærland T Pripp AH Diseth TH . Individual characteristics of students with autism spectrum disorders and school refusal behavior. Autism. (2019) 23:413–23. doi: 10.1177/1362361317748619 29241346

[41] Orm S Mebostad M Orm CB Løkke JA . Skolefravær og skolevegring hos elever med ADHD: en scoping review. Psykologi. i Kommunen. (2020) 4:4.

[42] McClemont AJ Morton HE Gillis JM Romanczyk RG . Brief report: predictors of school refusal due to bullying in children with autism spectrum disorder and attentiondeficit/hyperactivity disorder. J Autism Dev Disord. (2021) 51:1781–8. doi: 10.1007/s10803-020-04640-y 32767172

[43] Martin AJ . The role of ADHD in academic adversity: disentangling ADHD effects from other personal and contextual factors. School. Psychol Q. (2014) 29:395–408. doi: 10.1037/spq0000069 24820011

[44] Filippello P Buzzai C Messina G Mafodda AV Sorrenti L . School refusal in students with low academic performances and specific learning disorder: the role of self-esteem and perceived parental psychological control. Int J Disability. Dev Educ. (2020) 67:592–607. doi: 10.1080/1034912X.2019.1626006 37339054

[45] Filippello P Sorrenti L Buzzai C Costa S . Predicting risk of school refusal: examining the incremental role of trait EI beyond personality and emotion regulation. Psihologija. (2018) 51:51–67. doi: 10.2298/psi170526013f

[46] Gonzálvez C Díaz-Herrero Á Vicent M Sanmartín R Fernández-Sogorb A Ruiz-Esteban C . Affective profiles and anxiety or non-anxiety-related reasons for school refusal behavior: latent profile analysis in Spanish adolescents. Front Psychol. (2021) 12:666218. doi: 10.3389/fpsyg.2021.666218 33841291 PMC8027341

[47] Gonzálvez C Kearney CA Jiménez-Ayala CE Sanmartín R Vicent M Inglés CJ . Functional profiles of school refusal behavior and their relationship with depression, anxiety, and stress. Psychiatry Res. (2019) 275:331–8. doi: 10.1016/j.psychres.2019.03.035 30149271

[48] Fernández-Sogorb A García-Fernández JM Vicent M Sanmartín R Inglés CJ Gonzálvez C . Relationship between school refusal behavior and social functioning: a cluster analysis approach. Rev Psicodidáctica. (2018) 23:21–7. doi: 10.1016/j.psicod.2017.07.001 38826717

[49] Hannan S Davis E Morrison S Gueorguieva R Tolin DF . An open trial of intensive cognitive-behavioral therapy for school refusal. Evidence-Based Pract Child Adolesc Ment Health. (2019) 4:89–101. doi: 10.1080/23794925.2019.1575707 37339054

[50] McKay-Brown L McGrath R Dalton L Graham L Smith A Ring J . Reengagement with education: a multidisciplinary home-school-clinic approach developed in Australia for schoolrefusing youth. Cogn Behav Pract. (2019) 26:92–106. doi: 10.1016/j.cbpra.2018.08.003 38826717

[51] Bernstein GA Hektner JM Borchardt CM McMillan MH . Treatment of school refusal: one-year follow-up. J Am Acad Child Adolesc Psychiatry. (2001) 40:206–13. doi: 10.1097/00004583-200102000-00015 11211369

[52] Maynard BR Heyne D Brendel KE Bulanda JJ Thompson AM Pigott TD . Treatment for school refusal among children and adolescents: a systematic review and meta-analysis. Res Soc Work. Pract. (2018) 28:56–67. doi: 10.1177/1049731515598619

[53] Nuttall C Woods K . Effective intervention for school refusal behaviour. Educ Psychol Pract. (2013) 29:347–66. doi: 10.1080/02667363.2013.846849 37339054

[54] Achenbach TM . As others see us: clinical and research implications of cross-informant correlations for psychopathology. Curr Dir psychol Sci. (2006) 15:94–8. doi: 10.1111/j.0963-7214.2006.00414.x 40046247

[55] De Los Reyes A Augenstein TM Wang M Thomas SA Drabick DA Burgers DE . The validity of the multi-informant approach to assessing child and adolescent mental health. psychol Bull. (2015) 141:858–900. doi: 10.1037/a0038498 25915035 PMC4486608

[56] Kraemer HC Measelle JR Ablow JC Essex MJ Boyce WT Kupfer DJ . A new approach to integrating data from multiple informants in psychiatric assessment and research: mixing and matching contexts and perspectives. Am J Psychiatry. (2003) 160:1566–77. doi: 10.1176/appi.ajp.160.9.1566 12944328

[57] Classi P Milton D Ward S Sarsour K Johnston J . Social and emotional difficulties in children with ADHD and the impact on school attendance and healthcare utilization. Child Adolesc Psychiatry Ment Health. (2012) 6:33. doi: 10.1186/1753-2000-6-33 23035861 PMC3489829

[58] Faraone SV Banaschewski T Coghill D Zheng Y Biederman J Bellgrove MA . The world federation of ADHD international consensus statement: 208 evidence-based conclusions about the disorder. Neurosci Biobehav Rev. (2021) 128:789–818. doi: 10.1016/j.neubiorev.2021.01.022 33549739 PMC8328933

[59] Guillemin F Bombardier C Beaton D . Cross-cultural adaptation of health-related quality of life measures: literature review and proposed guidelines. J Clin Epidemiol. (1993) 46:1417–32. doi: 10.1016/0895-4356(93)90142-N 8263569

[60] Hambleton RK Zenisky AL . Translating and adapting tests for cross-cultural assessments. In: Matsumoto D van de Vijver FJR , editors. Cross-Cultural Research Methods in Psychology. Cambridge, UK: Cambridge University Press (2011). p. 46–74. doi: 10.1017/CBO9780511779381.004

[61] JASP Team . JASP. (2020), (version 0.13.1)[Dataset]. Amsterdam, The Netherlands: JASP Team.

[62] DiStefano C Morgan GB . A comparison of diagonal weighted least squares robust estimation techniques for ordinal data. Struct Equation. Model.: A Multidiscip J. (2014) 21:425–38. doi: 10.1080/10705511.2014.915373 37339054

[63] Brown TA . Confirmatory Factor Analysis for Applied Research. 2nd edn. New York, NY: Guilford Press (2015).

[64] Flora DB Curran PJ . An empirical evaluation of alternative methods of estimation for confirmatory factor analysis with ordinal data. psychol Methods. (2004) 9:466–91. doi: 10.1037/1082-989X.9.4.466 15598100 PMC3153362

[65] Li C-H . Confirmatory factor analysis with ordinal data: comparing robust maximum likelihood and diagonally weighted least squares. Behav Res Methods. (2016) 48:936–49. doi: 10.3758/s13428-015-0619-7 26174714

[66] Mueller RO Hancock GR . Factor analysis and latent structure, confirmatory. In: Smelser NJ Baltes PB , editors. International Encyclopedia of the Social and Behavioral Sciences. Pergamon (2001). p. 5239–44. doi: 10.1016/B0-08-043076-7/00426-5

[67] Alhija FN-A . Factor analysis: an overview and some contemporary advances. In: Peterson P Baker E McGaw B , editors. International Encyclopedia of Education, 3rd edn. Oxford, UK: Elsevier, Amsterdam (2010). p. 162–70. doi: 10.1016/B978-0-08-044894-7.01328-2

[68] Hu L Bentler PM . Cutoff criteria for fit indexes in covariance structure analysis: conventional criteria versus new alternatives. Struct Equation. Model.: A Multidiscip J. (1999) 6:1–55. doi: 10.1080/10705519909540118 37339054

[69] Cohen J . Statistical Power Analysis for the Behavioral Sciences. 2nd edn. Hillsdale, NJ: Lawrence Erlbaum Associates (1988).

[70] Nunnally JC Bernstein IH . Psychometric Theory. New York: McGraw-Hill (1994). 3rd edn.

[71] McNeish D . Thanks coefficient alpha, we’ll take it from here. psychol Methods. (2018) 23:412–33. doi: 10.1037/met0000144 28557467

[72] Revelle W Zinbarg RE . Coefficients alpha, beta, omega, and the GLB: comments on Sijtsma. Psychometrika. (2009) 74:145–54. doi: 10.1007/s11336-008-9102-z 30311153

[73] Dunn TJ Baguley T Brunsden V . From alpha to omega: a practical solution to the pervasive problem of internal consistency estimation. Br J Psychol. (2014) 105:399–412. doi: 10.1111/bjop.12046 24844115

[74] Trizano-Hermosilla Í Alvarado JM . Best alternatives to Cronbach’s alpha reliability in realistic conditions: congeneric and asymmetrical measurements. Front Psychol. (2016) 7:769. doi: 10.3389/fpsyg.2016.00769 27303333 PMC4880791

[75] Shrout PE Fleiss JL . Intraclass correlations: uses in assessing rater reliability. psychol Bull. (1979) 86:420–8. doi: 10.1037/0033-2909.86.2.420 18839484

[76] McGraw KO Wong SP . Forming inferences about some intraclass correlation coefficients. psychol Methods. (1996) 1:30–46. doi: 10.1037/1082-989X.1.1.30 27371692

[77] Koo TK Li MY . A guideline of selecting and reporting intraclass correlation coefficients for reliability research. J Chiropractic. Med. (2016) 15:155–63. doi: 10.1016/j.jcm.2016.02.012 27330520 PMC4913118

[78] Cicchetti DV . Guidelines, criteria, and rules of thumb for evaluating normed and standardized assessment instruments in psychology. psychol Assess. (1994) 6:284–90. doi: 10.1037/1040-3590.6.4.284 27371692

[79] Lu W . Adolescent depression: national trends, risk factors, and healthcare disparities. Am J Health Behav. (2019) 43:181–94. doi: 10.5993/AJHB.43.1.15 30522576

[80] Tekin İ Aydın S . School refusal and anxiety among children and adolescents: a systematic scoping review. New Dir For. Child Adolesc Dev. (2022) 2022:43–65. doi: 10.1002/cad.20481 36161758

[81] Beidel DC Turner SM . Childhood Anxiety Disorders: A Guide to Research and Treatment. New York: Routledge (2005). doi: 10.4324/9780203956014

[82] Heyne D King NJ Tonge BJ Rollings S Young D Pritchard M . Evaluation of child therapy and caregiver training in the treatment of school refusal. J Am Acad Child Adolesc Psychiatry. (2002) 41:687–95. doi: 10.1097/00004583-200206000-00008 12049443

[83] Bernstein GA Borchardt CM Perwien AR Crosby RD Kushner MG Thuras PD . Imipramine plus cognitive-behavioral therapy in the treatment of school refusal. J Am Acad Child Adolesc Psychiatry. (2000) 39:276–83. doi: 10.1097/00004583-200003000-00008 10714046

[84] Kearney CA . Forms and functions of school refusal behavior in youth: an empirical analysis of absenteeism severity. J Child Psychol Psychiatry. (2007) 48:53–61. doi: 10.1111/j.1469-7610.2006.01634.x 17244270

[85] Havik T Bru E Ertesvåg SK . School factors associated with school refusal- and truancy-related reasons for school non-attendance. Soc Psychol Educ. (2015) 18:221–40. doi: 10.1007/s11218-015-9293-y 30311153

[86] Fornander MJ Kearney CA . Internalizing symptoms as predictors of school absenteeism severity at multiple levels: ensemble and classification and regression tree analysis. Front Psychol. (2020) 10:3079. doi: 10.3389/fpsyg.2019.03079 32038423 PMC6985447

[87] Kearney CA Bensaheb A . School absenteeism and school refusal behavior: a review and suggestions for school-based health professionals. J School. Health. (2006) 76:3–7. doi: 10.1111/j.1746-1561.2006.00060.x 16457678

[88] Birmaher B Khetarpal S Brent D Cully M Balach L Kaufman J . The Screen for Child Anxiety Related Emotional Disorders (SCARED): scale construction and psychometric characteristics. J Am Acad Child Adolesc Psychiatry. (1997) 36:545–53. doi: 10.1097/00004583-199704000-00018 9100430

[89] March JS Parker JD Sullivan K Stallings P Conners CK . The Multidimensional Anxiety Scale for Children (MASC): factor structure, reliability, and validity. J Am Acad Child Adolesc Psychiatry. (1997) 36:554–65. doi: 10.1097/00004583-199704000-00019 9100431

[90] Kovacs M . Children’s Depression Inventory (Cdi2): Technical Manual. North Tonawanda, NY: Multi-Health Systems (2011).

[91] Kovacs M . Rating scales to assess depression in school-aged children. Acta Paedopsychiatrica. (1981) 46:305–15. 7025571

[92] Radloff LS . The CES-D Scale: a self-report depression scale for research in the general population. Appl psychol Measurement. (1977) 1:385–401. doi: 10.1177/014662167700100306 26918431

